# Crystal structure of ethyl 2-phenyl-9-phenyl­sulfonyl-9*H*-carbazole-3-carboxyl­ate

**DOI:** 10.1107/S205698901501662X

**Published:** 2015-09-12

**Authors:** M. Umadevi, P. Raju, R. Yamuna, A. K. Mohanakrishnan, G. Chakkaravarthi

**Affiliations:** a Research and Development Centre, Bharathiar University, Coimbatore 641 046, India; b Department of Chemistry, Pallavan College of Engineering, Kanchipuram 631 502, India; c Department of Organic Chemistry, University of Madras, Guindy Campus, Chennai 600 025, India; d Department of Sciences, Chemistry and Materials Research Lab, Amrita Vishwa Vidyapeetham University, Ettimadai, Coimbatore 641 112, India; e Department of Physics, CPCL Polytechnic College, Chennai 600 068, India

**Keywords:** crystal structure, ester, phenyl­sulfon­yl, 9*H*-carbazole-3-carboxyl­ate, biological activity, indole derivatives, hydrogen bonding, C—H⋯π inter­actions, π–π inter­actions

## Abstract

In the title compound, C_27_H_21_NO_4_S, the dihedral angles between the carbazole ring system (r.m.s. deviation = 0.015 Å) and the sulfur-bonded and directly linked benzene rings are 79.98 (11) and 53.51 (18)°, respectively. The benzene rings subtend a dihedral angle of 48.4 (2)°. The ethyl side chain of the ester group has an extended conformation [C—O—C—C = −172.3 (3)°]. In the crystal, inversion dimers linked by pairs of weak C—H⋯O hydrogen bonds generate *R*_2_^2^(22) loops. The dimers are linked by weak C—H⋯π and π–π [centroid-to-centroid distances ranging from 3.5042 (14) to 3.888 (2) Å] inter­actions, thereby forming a three-dimensional supra­molecular network.

## Related literature   

For the biological activity of indole derivatives, see: Itoigawa *et al.* (2000 ▸); Ramsewak *et al.* (1999 ▸). For related structures, see: Chakkaravarthi *et al.* (2008 ▸, 2009 ▸).
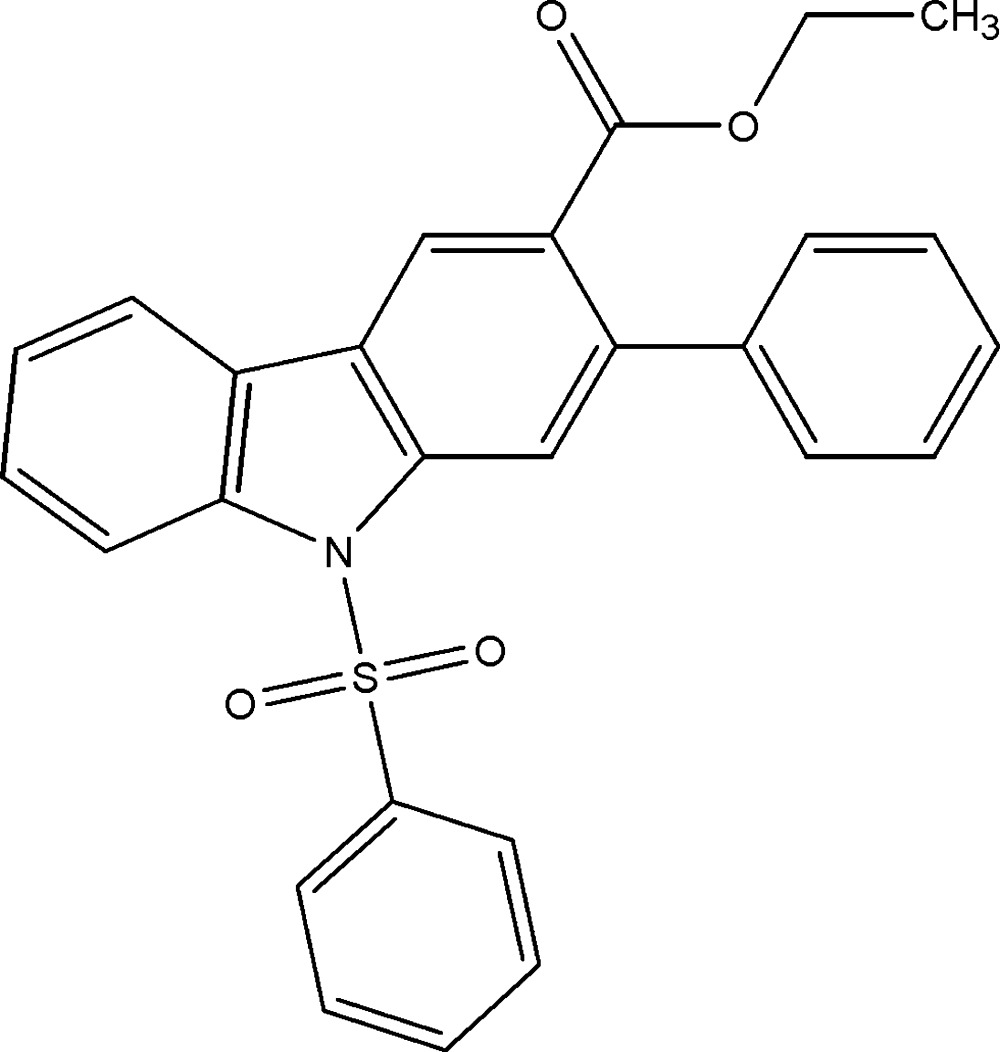


## Experimental   

### Crystal data  

C_27_H_21_NO_4_S
*M* *_r_* = 455.51
Monoclinic, 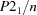

*a* = 13.7655 (8) Å
*b* = 7.8207 (4) Å
*c* = 20.9500 (12) Å
β = 94.054 (2)°
*V* = 2249.7 (2) Å^3^
*Z* = 4
Mo *K*α radiation
μ = 0.18 mm^−1^
*T* = 295 K
0.28 × 0.26 × 0.24 mm

### Data collection  

Bruker Kappa APEXII CCD diffractometer
Absorption correction: multi-scan (*SADABS*; Sheldrick, 1996 ▸) *T*_min_ = 0.952, *T*_max_ = 0.958
39017 measured reflections
4997 independent reflections
2939 reflections with *I* > 2σ(*I*)
*R*_int_ = 0.048

### Refinement  

*R*[*F* ^2^ > 2σ(*F* ^2^)] = 0.048
*wR*(*F* ^2^) = 0.146
*S* = 1.04
4997 reflections
299 parameters
4 restraints
H-atom parameters constrained
Δρ_max_ = 0.19 e Å^−3^
Δρ_min_ = −0.34 e Å^−3^

### 

Data collection: *APEX2* (Bruker, 2004 ▸); cell refinement: *SAINT* (Bruker, 2004 ▸); data reduction: *SAINT*; program(s) used to solve structure: *SHELXS97* (Sheldrick, 2008 ▸); program(s) used to refine structure: *SHELXL97* (Sheldrick, 2008 ▸); molecular graphics: *PLATON* (Spek, 2009 ▸); software used to prepare material for publication: *SHELXL97* and *PLATON*.

## Supplementary Material

Crystal structure: contains datablock(s) global, I. DOI: 10.1107/S205698901501662X/hb7499sup1.cif

Structure factors: contains datablock(s) I. DOI: 10.1107/S205698901501662X/hb7499Isup2.hkl

Supporting information file. DOI: 10.1107/S205698901501662X/hb7499Isup3.cml

**Fig. 1.**
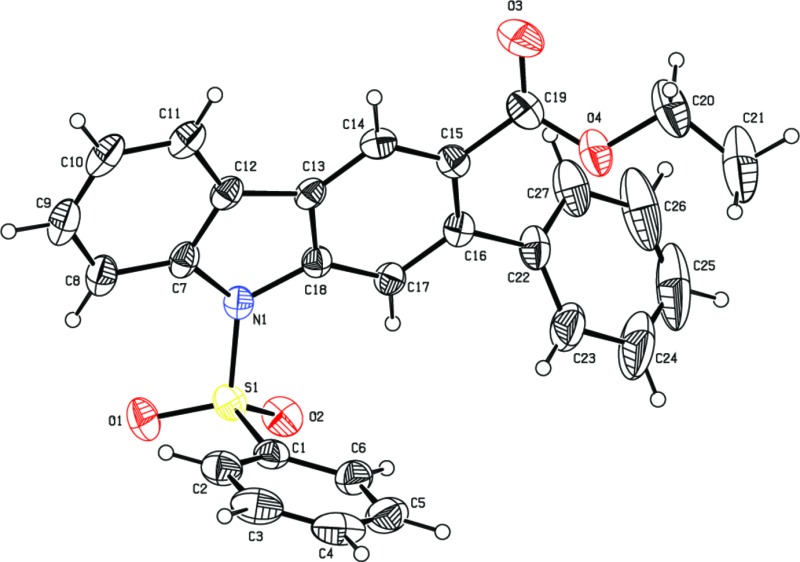
The molecular structure of (I), with 30% probability displacement ellipsoids for non-H atoms.

**Fig. 2.**
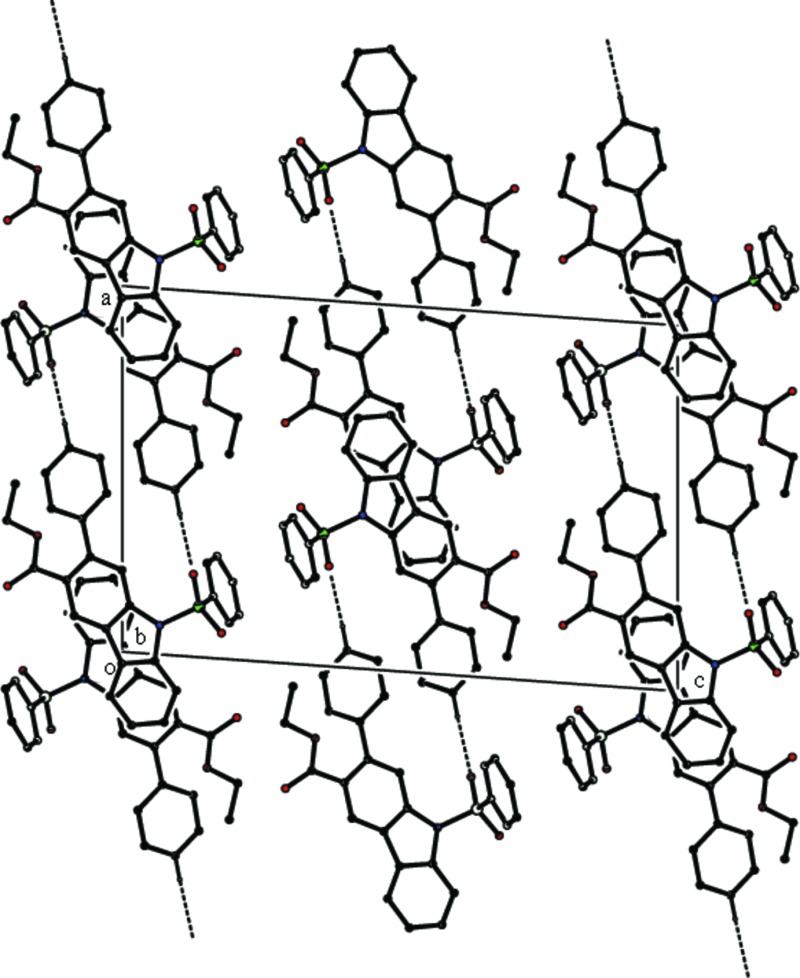
The crystal packing of the title compound viewed along the b axis. The hydrogen bonds are shown as dashed lines (see Table 1), and C-bound H atoms have been omitted for clarity.

. DOI: 10.1107/S205698901501662X/hb7499fig1.tif
The mol­ecular structure of (I), with 30% probability displacement ellipsoids for non-H atoms.

b . DOI: 10.1107/S205698901501662X/hb7499fig2.tif
The crystal packing of the title compound viewed along the *b* axis. The hydrogen bonds are shown as dashed lines (see Table 1), and C-bound H atoms have been omitted for clarity.

CCDC reference: 1422541

Additional supporting information:  crystallographic information; 3D view; checkCIF report

## Figures and Tables

**Table 1:** Hydrogen-bond geometry (, ) *Cg*2 is the centroid of the C1C6 ring.

*D*H*A*	*D*H	H*A*	*D* *A*	*D*H*A*
C25H25O2^i^	0.93	2.53	3.439(5)	166
C11H11*Cg*2^ii^	0.93	2.83	3.752(3)	172

Symmetry codes: (i) 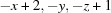; (ii) 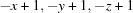.

## supplementary crystallographic information

### S1. Comment

Carbazole and its derivatives have become quite attractive compounds owing to their applications in pharmacy and molecular electronics. It has been reported that carbazole derivatives exhibit various biological activities such as antitumor (Itoigawa, *et al.* 2000), anti-inflammatory and antimutagenic (Ramsewak, *et al.* 1999). The molecular structure of the title compound (I) is shown in Fig. 1. The geometric parameters of (I) are comparable with the similar structures [Chakkaravarthi *et al.* 2008, 2009].

In the crystal, a pair of C—H···O hydrogen bonds generates *R*_2_^2^(22)graph-set motif and the crystal packing is also influenced by weak C—H···π (Table 1) and π–π [*Cg*1···*Cg*1^i^ distance 3.5042 (14) Å; *Cg*1···*Cg*3^i^ distance 3.8449 (15) Å; *Cg*3···*Cg*4^i^ distance 3.8882 (15) Å (i) 1 - *x*,-*y*,1 - *z*; *Cg*1, *Cg*2 and *Cg*3 are the centroids of the rings (N1/C7/C12/C13/C18), (C1—C6) and (C7—C12) respectively] interactions, forming a three-dimensional network.

### S2. Experimental

The thermal electrocyclization of 3-(1-(phenylsulfonyl)-2-styryl -1*H*-indol-3-yl) acrylate (0.25 g) in dry xylenes (10 ml), 10% Pd/C (0.08 g) was added and the reaction mixture was refluxed for 12 h. After completion of the reaction (monitored by TLC), the reaction mass was filtered through celite bed and washed with hot xylenes (10 ml). The combined filtrate was evaporated under reduced presser followed by tituration of the resulting crude product with methanol afforded the title compound as a colourless solid.

### S3. Refinement

H atoms were positioned geometrically and refined using riding model, with C—H = 0.93 Å and *U*_iso_(H) = 1.2Ueq(C) for C—H, C—H = 0.97 Å and *U*_iso_(H) = 1.2Ueq(C) for CH_2_ and C—H = 0.96 Å and *U*_iso_(H) = 1.5Ueq(C) for CH_3_. The anisotropic displacement parameters were restrained within 0.001 using DELU command in the direction of C24—C25, C25—C26 and S1—O2 bonds.

### Crystal data

**Table:**

C_27_H_21_NO_4_S	*F*(000) = 952
*M**_r_* = 455.51	*D*_x_ = 1.345 Mg m^−^^3^
Monoclinic, *P*2_1_/*n*	Mo *K*α radiation, λ = 0.71073 Å
Hall symbol: -P 2yn	Cell parameters from 9043 reflections
*a* = 13.7655 (8) Å	θ = 2.8–24.7°
*b* = 7.8207 (4) Å	µ = 0.18 mm^−^^1^
*c* = 20.9500 (12) Å	*T* = 295 K
β = 94.054 (2)°	Block, colourless
*V* = 2249.7 (2) Å^3^	0.28 × 0.26 × 0.24 mm
*Z* = 4	

### Data collection

**Table:**

Bruker Kappa APEXII CCD diffractometer	4997 independent reflections
Radiation source: fine-focus sealed tube	2939 reflections with *I* > 2σ(*I*)
Graphite monochromator	*R*_int_ = 0.048
ω and φ scan	θ_max_ = 27.3°, θ_min_ = 2.8°
Absorption correction: multi-scan (*SADABS*; Sheldrick, 1996)	*h* = −17→17
*T*_min_ = 0.952, *T*_max_ = 0.958	*k* = −9→9
39017 measured reflections	*l* = −26→26

### Refinement

**Table:**

Refinement on *F*^2^	Primary atom site location: structure-invariant direct methods
Least-squares matrix: full	Secondary atom site location: difference Fourier map
*R*[*F*^2^ > 2σ(*F*^2^)] = 0.048	Hydrogen site location: inferred from neighbouring sites
*wR*(*F*^2^) = 0.146	H-atom parameters constrained
*S* = 1.04	*w* = 1/[σ^2^(*F*_o_^2^) + (0.0543*P*)^2^ + 1.1538*P*] where *P* = (*F*_o_^2^ + 2*F*_c_^2^)/3
4997 reflections	(Δ/σ)_max_ < 0.001
299 parameters	Δρ_max_ = 0.19 e Å^−^^3^
4 restraints	Δρ_min_ = −0.34 e Å^−^^3^

### Special details

**Table:**

Geometry. All e.s.d.'s (except the e.s.d. in the dihedral angle between two l.s. planes) are estimated using the full covariance matrix. The cell e.s.d.'s are taken into account individually in the estimation of e.s.d.'s in distances, angles and torsion angles; correlations between e.s.d.'s in cell parameters are only used when they are defined by crystal symmetry. An approximate (isotropic) treatment of cell e.s.d.'s is used for estimating e.s.d.'s involving l.s. planes.
Refinement. Refinement of *F*^2^ against ALL reflections. The weighted *R*-factor *wR* and goodness of fit *S* are based on *F*^2^, conventional *R*-factors *R* are based on *F*, with *F* set to zero for negative *F*^2^. The threshold expression of *F*^2^ > σ(*F*^2^) is used only for calculating *R*-factors(gt) *etc*. and is not relevant to the choice of reflections for refinement. *R*-factors based on *F*^2^ are statistically about twice as large as those based on *F*, and *R*- factors based on ALL data will be even larger.

### Fractional atomic coordinates and isotropic or equivalent isotropic displacement parameters (Å^2^)

**Table:**

	*x*	*y*	*z*	*U*_iso_*/*U*_eq_	
C1	0.66860 (19)	0.3090 (3)	0.66383 (11)	0.0483 (6)	
C2	0.6003 (2)	0.4052 (4)	0.69291 (13)	0.0620 (7)	
H2	0.5399	0.3594	0.7007	0.074*	
C3	0.6240 (3)	0.5717 (4)	0.71020 (15)	0.0806 (10)	
H3	0.5791	0.6392	0.7298	0.097*	
C4	0.7125 (3)	0.6369 (4)	0.69873 (15)	0.0834 (11)	
H4	0.7273	0.7492	0.7103	0.100*	
C5	0.7798 (3)	0.5405 (5)	0.67054 (16)	0.0807 (10)	
H5	0.8403	0.5869	0.6634	0.097*	
C6	0.7586 (2)	0.3740 (4)	0.65255 (13)	0.0625 (7)	
H6	0.8041	0.3074	0.6332	0.075*	
C7	0.48068 (17)	0.1830 (3)	0.55839 (12)	0.0469 (6)	
C8	0.4049 (2)	0.1610 (3)	0.59744 (14)	0.0597 (7)	
H8	0.4138	0.1059	0.6367	0.072*	
C9	0.3155 (2)	0.2248 (4)	0.57535 (17)	0.0699 (9)	
H9	0.2632	0.2133	0.6007	0.084*	
C10	0.3011 (2)	0.3051 (4)	0.51685 (17)	0.0708 (9)	
H10	0.2395	0.3462	0.5036	0.085*	
C11	0.37638 (19)	0.3250 (4)	0.47802 (15)	0.0600 (7)	
H11	0.3667	0.3787	0.4385	0.072*	
C12	0.46766 (17)	0.2630 (3)	0.49929 (12)	0.0470 (6)	
C13	0.55983 (17)	0.2628 (3)	0.47067 (11)	0.0426 (6)	
C14	0.58915 (19)	0.3254 (3)	0.41368 (12)	0.0497 (6)	
H14	0.5440	0.3765	0.3846	0.060*	
C15	0.68539 (19)	0.3126 (3)	0.39965 (11)	0.0475 (6)	
C16	0.75333 (18)	0.2285 (3)	0.44225 (12)	0.0466 (6)	
C17	0.72340 (17)	0.1631 (3)	0.49910 (11)	0.0444 (6)	
H17	0.7673	0.1061	0.5274	0.053*	
C18	0.62778 (17)	0.1835 (3)	0.51332 (11)	0.0416 (5)	
C19	0.7118 (2)	0.4012 (3)	0.34099 (14)	0.0607 (7)	
C20	0.8370 (3)	0.5489 (6)	0.2923 (2)	0.1093 (14)	
H20A	0.8004	0.6526	0.2822	0.131*	
H20B	0.8302	0.4733	0.2556	0.131*	
C21	0.9384 (4)	0.5893 (7)	0.3073 (3)	0.157 (2)	
H21A	0.9443	0.6653	0.3434	0.236*	
H21B	0.9644	0.6434	0.2711	0.236*	
H21C	0.9739	0.4860	0.3175	0.236*	
C22	0.8555 (2)	0.1969 (4)	0.42791 (15)	0.0608 (7)	
C23	0.9301 (2)	0.2432 (6)	0.4718 (2)	0.0980 (12)	
H23	0.9162	0.2978	0.5096	0.118*	
C24	1.0256 (3)	0.2082 (8)	0.4597 (3)	0.158 (2)	
H24	1.0756	0.2380	0.4898	0.190*	
C25	1.0471 (4)	0.1317 (8)	0.4049 (4)	0.173 (3)	
H25	1.1117	0.1126	0.3966	0.208*	
C26	0.9744 (4)	0.0826 (6)	0.3620 (3)	0.147 (2)	
H26	0.9895	0.0263	0.3248	0.176*	
C27	0.8780 (3)	0.1146 (4)	0.37239 (18)	0.0861 (11)	
H27	0.8287	0.0811	0.3424	0.103*	
N1	0.58011 (14)	0.1277 (2)	0.56748 (9)	0.0444 (5)	
O1	0.57068 (14)	0.0310 (2)	0.67859 (9)	0.0702 (6)	
O2	0.72574 (14)	0.0126 (2)	0.62797 (9)	0.0634 (5)	
O3	0.65793 (19)	0.4200 (3)	0.29378 (10)	0.0948 (8)	
O4	0.80077 (15)	0.4669 (3)	0.34730 (10)	0.0734 (6)	
S1	0.63858 (5)	0.10114 (8)	0.63854 (3)	0.0509 (2)	

### Atomic displacement parameters (Å^2^)

**Table:**

	*U*^11^	*U*^22^	*U*^33^	*U*^12^	*U*^13^	*U*^23^
C1	0.0547 (16)	0.0481 (14)	0.0409 (13)	0.0008 (12)	−0.0055 (11)	0.0048 (11)
C2	0.0679 (19)	0.0637 (17)	0.0538 (16)	0.0102 (15)	0.0008 (14)	−0.0075 (14)
C3	0.109 (3)	0.068 (2)	0.0622 (19)	0.019 (2)	−0.0103 (19)	−0.0144 (16)
C4	0.132 (3)	0.0536 (18)	0.0597 (19)	−0.006 (2)	−0.030 (2)	0.0012 (15)
C5	0.089 (3)	0.078 (2)	0.072 (2)	−0.029 (2)	−0.0194 (18)	0.0139 (18)
C6	0.0603 (19)	0.0667 (18)	0.0591 (17)	−0.0064 (14)	−0.0056 (14)	0.0076 (14)
C7	0.0411 (15)	0.0379 (12)	0.0624 (16)	−0.0072 (11)	0.0091 (12)	−0.0121 (11)
C8	0.0522 (18)	0.0551 (15)	0.0735 (18)	−0.0113 (13)	0.0173 (14)	−0.0106 (14)
C9	0.0479 (19)	0.0633 (18)	0.101 (3)	−0.0128 (14)	0.0230 (17)	−0.0223 (18)
C10	0.0380 (17)	0.0687 (19)	0.105 (3)	−0.0051 (14)	−0.0003 (17)	−0.0231 (18)
C11	0.0417 (16)	0.0593 (16)	0.0779 (19)	−0.0003 (13)	−0.0036 (14)	−0.0114 (14)
C12	0.0425 (15)	0.0393 (12)	0.0587 (15)	−0.0049 (11)	0.0016 (12)	−0.0124 (11)
C13	0.0383 (14)	0.0390 (12)	0.0503 (14)	−0.0034 (10)	0.0006 (11)	−0.0076 (11)
C14	0.0518 (16)	0.0478 (13)	0.0485 (14)	0.0027 (12)	−0.0038 (12)	−0.0051 (11)
C15	0.0537 (16)	0.0427 (13)	0.0464 (14)	0.0003 (12)	0.0064 (12)	−0.0030 (11)
C16	0.0444 (15)	0.0412 (12)	0.0550 (15)	−0.0004 (11)	0.0078 (12)	−0.0024 (11)
C17	0.0397 (14)	0.0450 (13)	0.0484 (14)	0.0038 (11)	0.0029 (11)	0.0005 (11)
C18	0.0438 (15)	0.0358 (11)	0.0454 (13)	−0.0048 (10)	0.0055 (11)	−0.0058 (10)
C19	0.074 (2)	0.0547 (16)	0.0545 (17)	0.0057 (15)	0.0150 (15)	0.0020 (13)
C20	0.124 (4)	0.106 (3)	0.105 (3)	−0.003 (3)	0.052 (3)	0.041 (2)
C21	0.121 (4)	0.149 (4)	0.212 (6)	−0.001 (3)	0.091 (4)	0.068 (4)
C22	0.0500 (17)	0.0580 (16)	0.0767 (19)	0.0079 (13)	0.0211 (15)	0.0163 (14)
C23	0.046 (2)	0.137 (3)	0.111 (3)	−0.007 (2)	0.006 (2)	0.036 (3)
C24	0.046 (3)	0.217 (6)	0.214 (5)	0.001 (3)	0.016 (3)	0.115 (4)
C25	0.089 (3)	0.160 (5)	0.284 (7)	0.056 (3)	0.106 (3)	0.131 (4)
C26	0.158 (4)	0.086 (3)	0.215 (5)	0.055 (3)	0.137 (4)	0.052 (3)
C27	0.094 (3)	0.0635 (19)	0.108 (3)	0.0209 (17)	0.056 (2)	0.0117 (18)
N1	0.0435 (12)	0.0426 (10)	0.0477 (11)	−0.0024 (9)	0.0078 (9)	−0.0016 (9)
O1	0.0807 (14)	0.0662 (12)	0.0660 (12)	−0.0102 (10)	0.0219 (11)	0.0172 (10)
O2	0.0687 (11)	0.0556 (11)	0.0662 (12)	0.0211 (9)	0.0060 (9)	0.0091 (9)
O3	0.113 (2)	0.115 (2)	0.0549 (13)	−0.0079 (15)	−0.0046 (13)	0.0193 (13)
O4	0.0736 (15)	0.0710 (13)	0.0786 (14)	0.0018 (11)	0.0273 (11)	0.0238 (11)
S1	0.0585 (4)	0.0428 (3)	0.0519 (4)	0.0026 (3)	0.0086 (3)	0.0075 (3)

### Geometric parameters (Å, º)

**Table:**

C1—C6	1.375 (4)	C15—C19	1.478 (4)
C1—C2	1.379 (4)	C16—C17	1.385 (3)
C1—S1	1.750 (3)	C16—C22	1.480 (3)
C2—C3	1.384 (4)	C17—C18	1.379 (3)
C2—H2	0.9300	C17—H17	0.9300
C3—C4	1.357 (5)	C18—N1	1.419 (3)
C3—H3	0.9300	C19—O3	1.202 (3)
C4—C5	1.361 (5)	C19—O4	1.326 (3)
C4—H4	0.9300	C20—O4	1.438 (4)
C5—C6	1.381 (4)	C20—C21	1.443 (6)
C5—H5	0.9300	C20—H20A	0.9700
C6—H6	0.9300	C20—H20B	0.9700
C7—C8	1.381 (3)	C21—H21A	0.9600
C7—C12	1.388 (4)	C21—H21B	0.9600
C7—N1	1.435 (3)	C21—H21C	0.9600
C8—C9	1.377 (4)	C22—C23	1.377 (5)
C8—H8	0.9300	C22—C27	1.383 (4)
C9—C10	1.379 (4)	C23—C24	1.385 (5)
C9—H9	0.9300	C23—H23	0.9300
C10—C11	1.371 (4)	C24—C25	1.345 (9)
C10—H10	0.9300	C24—H24	0.9300
C11—C12	1.391 (4)	C25—C26	1.354 (9)
C11—H11	0.9300	C25—H25	0.9300
C12—C13	1.441 (3)	C26—C27	1.383 (5)
C13—C14	1.377 (3)	C26—H26	0.9300
C13—C18	1.393 (3)	C27—H27	0.9300
C14—C15	1.381 (3)	N1—S1	1.655 (2)
C14—H14	0.9300	O1—S1	1.4103 (18)
C15—C16	1.409 (3)	O2—S1	1.4163 (18)
			
C6—C1—C2	121.6 (3)	C18—C17—H17	120.4
C6—C1—S1	119.2 (2)	C16—C17—H17	120.4
C2—C1—S1	119.1 (2)	C17—C18—C13	121.4 (2)
C1—C2—C3	118.3 (3)	C17—C18—N1	129.7 (2)
C1—C2—H2	120.9	C13—C18—N1	108.8 (2)
C3—C2—H2	120.9	O3—C19—O4	123.1 (3)
C4—C3—C2	120.3 (3)	O3—C19—C15	124.6 (3)
C4—C3—H3	119.8	O4—C19—C15	112.2 (3)
C2—C3—H3	119.8	O4—C20—C21	107.9 (4)
C3—C4—C5	121.1 (3)	O4—C20—H20A	110.1
C3—C4—H4	119.5	C21—C20—H20A	110.1
C5—C4—H4	119.5	O4—C20—H20B	110.1
C4—C5—C6	120.2 (3)	C21—C20—H20B	110.1
C4—C5—H5	119.9	H20A—C20—H20B	108.4
C6—C5—H5	119.9	C20—C21—H21A	109.5
C1—C6—C5	118.6 (3)	C20—C21—H21B	109.5
C1—C6—H6	120.7	H21A—C21—H21B	109.5
C5—C6—H6	120.7	C20—C21—H21C	109.5
C8—C7—C12	122.0 (2)	H21A—C21—H21C	109.5
C8—C7—N1	129.6 (3)	H21B—C21—H21C	109.5
C12—C7—N1	108.4 (2)	C23—C22—C27	119.0 (3)
C9—C8—C7	116.7 (3)	C23—C22—C16	119.6 (3)
C9—C8—H8	121.6	C27—C22—C16	121.4 (3)
C7—C8—H8	121.6	C22—C23—C24	119.9 (5)
C8—C9—C10	122.2 (3)	C22—C23—H23	120.1
C8—C9—H9	118.9	C24—C23—H23	120.1
C10—C9—H9	118.9	C25—C24—C23	120.9 (6)
C11—C10—C9	120.7 (3)	C25—C24—H24	119.5
C11—C10—H10	119.6	C23—C24—H24	119.5
C9—C10—H10	119.6	C24—C25—C26	119.7 (5)
C10—C11—C12	118.3 (3)	C24—C25—H25	120.1
C10—C11—H11	120.8	C26—C25—H25	120.1
C12—C11—H11	120.8	C25—C26—C27	121.1 (6)
C7—C12—C11	120.0 (2)	C25—C26—H26	119.4
C7—C12—C13	108.0 (2)	C27—C26—H26	119.4
C11—C12—C13	132.0 (3)	C22—C27—C26	119.4 (4)
C14—C13—C18	119.3 (2)	C22—C27—H27	120.3
C14—C13—C12	132.9 (2)	C26—C27—H27	120.3
C18—C13—C12	107.7 (2)	C18—N1—C7	106.97 (19)
C13—C14—C15	120.2 (2)	C18—N1—S1	122.35 (16)
C13—C14—H14	119.9	C7—N1—S1	123.77 (16)
C15—C14—H14	119.9	C19—O4—C20	117.6 (3)
C14—C15—C16	120.2 (2)	O1—S1—O2	120.49 (12)
C14—C15—C19	116.0 (2)	O1—S1—N1	106.47 (11)
C16—C15—C19	123.7 (2)	O2—S1—N1	106.51 (10)
C17—C16—C15	119.5 (2)	O1—S1—C1	109.47 (12)
C17—C16—C22	117.2 (2)	O2—S1—C1	108.49 (12)
C15—C16—C22	123.2 (2)	N1—S1—C1	104.18 (10)
C18—C17—C16	119.3 (2)		
			
C6—C1—C2—C3	0.7 (4)	C14—C15—C19—O3	−31.9 (4)
S1—C1—C2—C3	−177.4 (2)	C16—C15—C19—O3	152.3 (3)
C1—C2—C3—C4	−0.2 (4)	C14—C15—C19—O4	144.6 (2)
C2—C3—C4—C5	−0.4 (5)	C16—C15—C19—O4	−31.2 (3)
C3—C4—C5—C6	0.6 (5)	C17—C16—C22—C23	−54.3 (4)
C2—C1—C6—C5	−0.6 (4)	C15—C16—C22—C23	129.2 (3)
S1—C1—C6—C5	177.5 (2)	C17—C16—C22—C27	122.7 (3)
C4—C5—C6—C1	−0.1 (4)	C15—C16—C22—C27	−53.8 (4)
C12—C7—C8—C9	−1.0 (4)	C27—C22—C23—C24	0.5 (5)
N1—C7—C8—C9	−178.3 (2)	C16—C22—C23—C24	177.6 (3)
C7—C8—C9—C10	0.8 (4)	C22—C23—C24—C25	1.0 (7)
C8—C9—C10—C11	−0.2 (4)	C23—C24—C25—C26	−2.3 (9)
C9—C10—C11—C12	−0.3 (4)	C24—C25—C26—C27	2.1 (9)
C8—C7—C12—C11	0.5 (4)	C23—C22—C27—C26	−0.8 (5)
N1—C7—C12—C11	178.4 (2)	C16—C22—C27—C26	−177.8 (3)
C8—C7—C12—C13	−179.2 (2)	C25—C26—C27—C22	−0.5 (7)
N1—C7—C12—C13	−1.3 (2)	C17—C18—N1—C7	179.8 (2)
C10—C11—C12—C7	0.1 (4)	C13—C18—N1—C7	−2.9 (2)
C10—C11—C12—C13	179.7 (2)	C17—C18—N1—S1	28.0 (3)
C7—C12—C13—C14	−179.2 (2)	C13—C18—N1—S1	−154.65 (16)
C11—C12—C13—C14	1.2 (4)	C8—C7—N1—C18	−179.8 (2)
C7—C12—C13—C18	−0.5 (3)	C12—C7—N1—C18	2.6 (2)
C11—C12—C13—C18	179.9 (2)	C8—C7—N1—S1	−28.5 (3)
C18—C13—C14—C15	−1.3 (3)	C12—C7—N1—S1	153.85 (16)
C12—C13—C14—C15	177.3 (2)	O3—C19—O4—C20	−6.6 (4)
C13—C14—C15—C16	2.9 (3)	C15—C19—O4—C20	176.9 (3)
C13—C14—C15—C19	−173.1 (2)	C21—C20—O4—C19	−172.3 (3)
C14—C15—C16—C17	−1.8 (3)	C18—N1—S1—O1	−174.87 (17)
C19—C15—C16—C17	173.8 (2)	C7—N1—S1—O1	38.1 (2)
C14—C15—C16—C22	174.6 (2)	C18—N1—S1—O2	−45.1 (2)
C19—C15—C16—C22	−9.7 (4)	C7—N1—S1—O2	167.82 (18)
C15—C16—C17—C18	−0.8 (3)	C18—N1—S1—C1	69.47 (19)
C22—C16—C17—C18	−177.5 (2)	C7—N1—S1—C1	−77.6 (2)
C16—C17—C18—C13	2.4 (3)	C6—C1—S1—O1	152.0 (2)
C16—C17—C18—N1	179.5 (2)	C2—C1—S1—O1	−29.9 (2)
C14—C13—C18—C17	−1.4 (3)	C6—C1—S1—O2	18.7 (2)
C12—C13—C18—C17	179.7 (2)	C2—C1—S1—O2	−163.2 (2)
C14—C13—C18—N1	−179.00 (19)	C6—C1—S1—N1	−94.5 (2)
C12—C13—C18—N1	2.1 (2)	C2—C1—S1—N1	83.7 (2)

### Hydrogen-bond geometry (Å, º)

Cg2 is the centroid of the C1–C6 ring.

**Table:**

*D*—H···*A*	*D*—H	H···*A*	*D*···*A*	*D*—H···*A*
C25—H25···O2^i^	0.93	2.53	3.439 (5)	166
C11—H11···*Cg*2^ii^	0.93	2.83	3.752 (3)	172

Symmetry codes: (i) −*x*+2, −*y*, −*z*+1; (ii) −*x*+1, −*y*+1, −*z*+1.
