# Supplementary figures and images for: Crystal structure of ethyl 2-phenyl-9-phenyl­sulfonyl-9H-carbazole-3-carboxyl­ate
Source: Acta Crystallogr E Crystallogr Commun. 2015 Sep 12;71(Pt 10):o725–6. doi: 10.1107/S205698901501662X (PMC4647343; doi:10.1107/S205698901501662X)

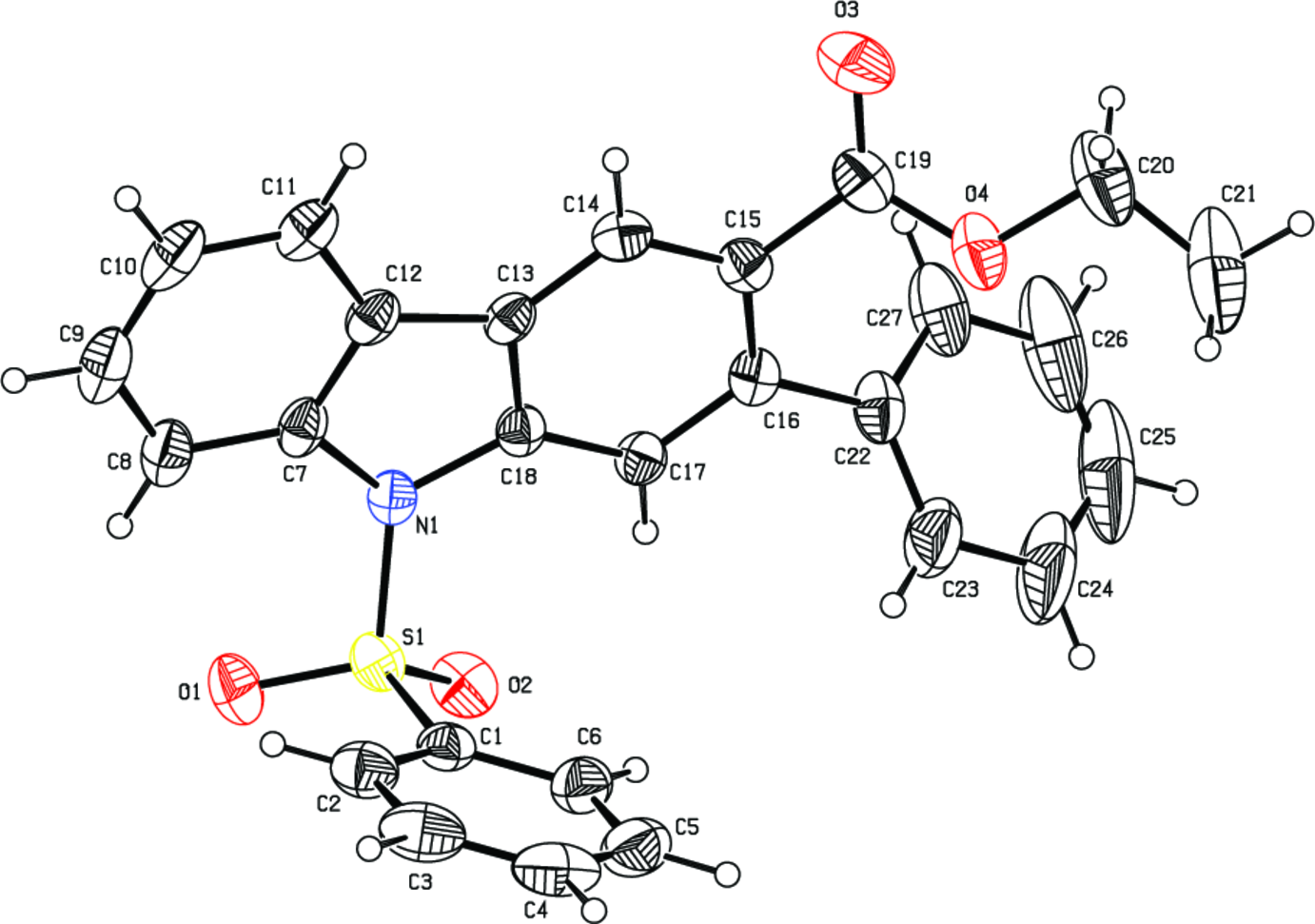

Supplement: Supplementary file 4 [file e-71-0o725-fig1.tif]

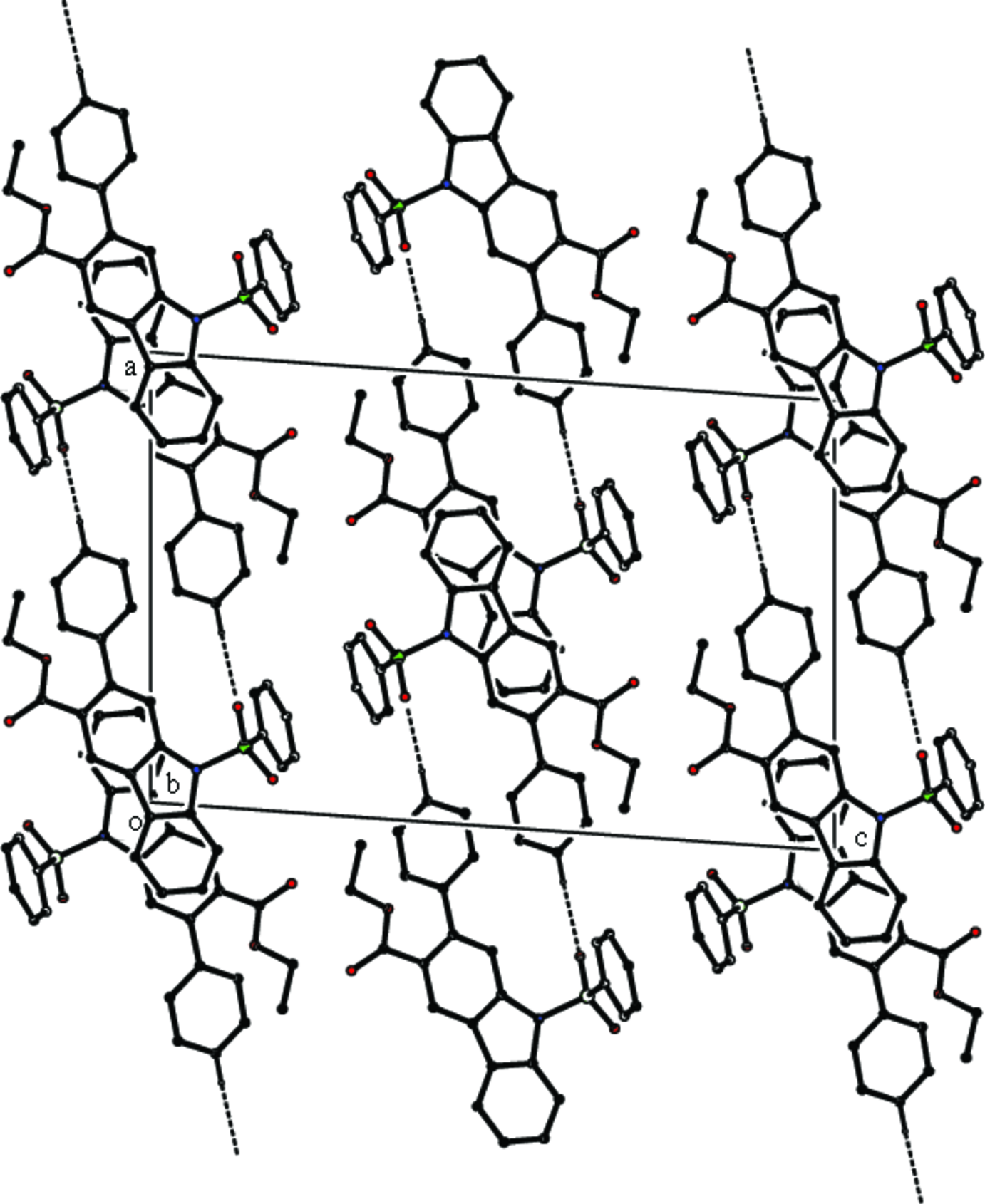

Supplement: Supplementary file 5 [file e-71-0o725-fig2.tif]
